# Metformin increases fasting glucose clearance and endogenous glucose production in non-diabetic individuals

**DOI:** 10.1007/s00125-019-05042-1

**Published:** 2019-11-22

**Authors:** Laura J. McCreight, Andrea Mari, Lucy Coppin, Nicola Jackson, A. Margot Umpleby, Ewan R. Pearson

**Affiliations:** 1 Division of Population Health and Genomics, University of Dundee, Ninewells Hospital, Dundee, DD19SY UK; 2 Institute of Neuroscience, National Research Council, Padova, Italy; 3 Faculty of Health and Medical Sciences, University of Surrey, Guildford, UK

**Keywords:** Endogenous glucose production, Glucose clearance, Metformin, Mixed-meal test, Stable isotopes

*To the Editor:* It is currently accepted that metformin inhibits complex I of the respiratory chain, altering the ratio of AMP or ADP to ATP and subsequently inhibiting gluconeogenesis [1, 2]. The original insulin clamp studies of the 1990s investigating the effect of metformin on glucose metabolism in obese and lean individuals with type 2 diabetes support this mechanism of action [3], demonstrating a reduction in endogenous glucose production (EGP) following metformin treatment. However, an increasing number of alternative pathways of metformin action have been proposed, including 5′ AMP-activated protein kinase (AMPK)-dependent and -independent effects, as well as gastrointestinal mechanisms of action [4, 5].

It is, therefore, with great interest that we read the article recently published in *Diabetologia* entitled ‘Metformin increases endogenous glucose production in non-diabetic individuals and individuals with recent-onset type 2 diabetes’ [6]. In the article, using studies of whole-body glucose metabolism, including non-steady state glucose tracer kinetics and hyperinsulinaemic–euglycaemic clamping, Gormsen et al report that metformin treatment is associated with an increase in the glucose rate of disappearance (*R*_d_), glucagon levels and EGP both in healthy control individuals and in individuals with recent-onset type 2 diabetes with good glycaemic control. These changes were absent in a placebo-treated group.

In this letter we present data from our recent study of non-diabetic individuals, which aimed to investigate the effect of metformin on glucose kinetics. Our findings corroborate the findings of Gormsen et al, adding to the pool of evidence indicating that the mechanism of action of metformin may be extra-hepatic.

Our study was conducted in the Clinical Research Centre at Ninewells Hospital, Dundee, between September 2016 and August 2017. It was co-sponsored by the University of Dundee and NHS Tayside, and ethical approval was granted by the East of Scotland Research Ethics Committee. The study was conducted in accordance with the Good Clinical Practice guidelines and the Declaration of Helsinki. The study was registered on the public database clinicaltrials.gov (identifier NCT02733679). Formal written informed consent was obtained from each individual prior to inclusion. Here we report the data from the non-diabetic controls.

In this non-randomised, non-blinded, open-label crossover study we treated non-diabetic individuals with two commonly used diabetes drugs, namely, metformin and pioglitazone. The time in study was 17 weeks, consisting of two 8 week treatment periods separated by 1 week washout. During treatment period one, participants were treated with metformin, titrated to a maximum of 1000 mg twice daily. During treatment period two, participants were treated with pioglitazone, titrated to 30 mg once daily. Each participant had three study visits: baseline, post-metformin and post-pioglitazone. Each visit included a dual-tracer mixed-meal test (MMT), to assess glucose kinetics in the fasted and non-steady state.

In brief, participants received a standardised meal the evening before the MMT. They were fasted from midnight and attended the Clinical Research Centre at 08:00 hours. After taking a baseline blood sample from each participant, a primed (6 mg/kg infused for 1 min) continuous (0.06 mg kg^−1^ min^−1^ for 120 min) infusion of [6,6-^2^H_2_]glucose (Euriso-Top, Saint-Aubin, France) was administered over an initial 2 h period, from −120 to 0 min, to reach steady state. Once in steady state, a liquid mixed meal labelled with [U-^13^C]glucose (1.7 g added to 71.9 g glucose drink, 220 ml total volume; Cambridge Isotope Laboratories, Andover, MA, USA), along with liquid paracetamol (1 g in 20 ml), was ingested. Over the following 6 h, a variable infusion of [6,6-^2^H_2_]glucose was administered, with the rate mimicking the expected EGP post meal. Blood samples were taken at 21 time points over the 8 h period. Samples were analysed for glucose at each time point, using gas chromatography–mass spectrometry to differentiate endogenous from labelled glucose (Agilent 7890A Gas Chromatographer and Agilent 5975 Mass Spectrometry detector, Agilent Technologies, Wokingham, UK). Insulin (ELISA 80-INSHU-E01.1, Alpco, Salem, MA, USA), glucagon (radioimmunoassay RIA; GL-32 K, Millipore, UK) and paracetamol level (enzymatic bichromatic endpoint technique on a Dimension VISTA 1500, Siemens; used as a measure of gastric emptying) were also measured. Glucose kinetics were modelled using the circulatory model [7], to assess EGP, glucose clearance and the rate of appearance of oral glucose. Statistical analyses were performed using R studio (rstudio.com; R version 3.1.0, R Foundation for Statistical Computing, Vienna, Austria). Variables are reported as fasting or meal mean (whereby the average increment was calculated by dividing the AUC of the variable in question by the duration of the meal test). Data were tested for normality using Shapiro–Wilk test. Normally distributed data are presented as mean ± SEM, and compared within group using the paired *t* test. Non-normally distributed data are presented as median with interquartile range (IQR) and compared using the Mann–Whitney *U* test. Statistical significance was defined as *p* < 0.05.

In total, 18 non-diabetic individuals were recruited. Of these, 15 completed the study, six of whom (40%) were female. The mean age was 22.4 years, BMI was 24.0 kg/m^2^, and HbA_1c_ was 33 mmol/mol (5.2%). One individual withdrew from the study due to metformin side effects, and two participants withdrew due to vomiting during the MMT.

Metformin did not affect gastric emptying, nor did it impact upon the rate of appearance of oral glucose. Fasting glucose and insulin concentrations were unchanged from baseline, following metformin treatment. However, there was an increase in fasting glucose clearance compared with baseline (baseline 2.7 [IQR 2.3–2.8] vs post metformin 3.0 [IQR 2.7–3.4] ml min^−1^ kg^−1^, *p*< 0.001). This was associated with an increase in the mean glucagon during the MMT (baseline 54.5 [IQR 35.8–71.0] vs post metformin 58.4 [IQR 42.3–78.4] pg/ml, *p* = 0.007). Fasting glucagon was elevated from baseline, though did not reach significance (baseline 54.8 [IQR 42.0–70.5] vs post metformin 65.2 [IQR 36.9–83.4] pg/ml, *p* = 0.28).

During fasting, participants are in steady state, where EGP is equal to *R*_d_. Therefore, as there is an increase in glucose clearance, metformin treatment also resulted in an increase in EGP under fasting conditions (baseline 10.9 [IQR 10.1–12.4] vs post metformin 13.8 [11.7–15.0] μmol min^−1^ kg^−1^, *p* < 0.001) (Fig. 1). Mean EGP during the MMT was elevated but did not reach significance (baseline 5.2 [IQR 4.8–6.5] vs post metformin 6.1 [IQR 4.4–7.0] μmol min^−1^ kg^−1^, *p* = 0.28). Pioglitazone had no impact on glucose clearance, EGP or glucagon.

**Fig. 1:**
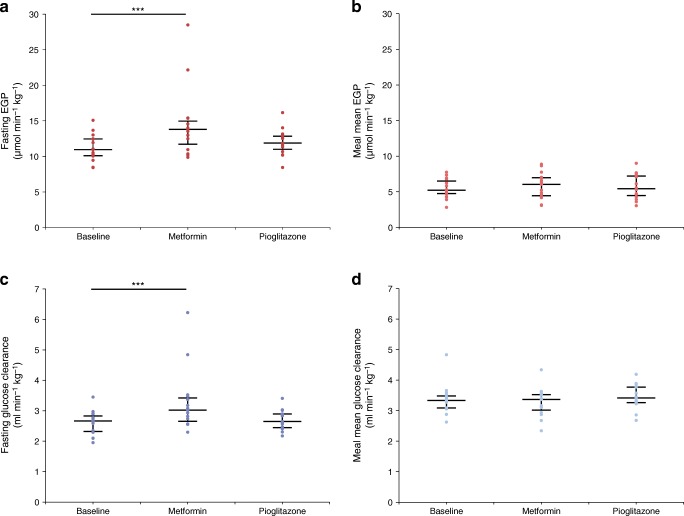
Fasting EGP (**a**); meal mean EGP (**b**); fasting glucose clearance (**c**) and meal mean glucose clearance (**d**) at all visits. Data are median (IQR) of all participants (*n*=15) in each cohort

This data from dual-tracer MMTs has provided some interesting insight into the mechanism of action of metformin. Metformin treatment was associated with increased fasting glucose clearance, which has been previously reported in clamp data [3, 8–10]. The increase in clearance could represent increased glucose uptake by the intestine, which has recently been postulated as a ‘glucose sink’ [11, 12].

Our non-diabetic group showed a metformin-related counter-regulatory increase in their mean glucagon level, and perhaps this occurs to prevent hypoglycaemia in the context of increased glucose clearance in these normoglycaemic individuals. In keeping with this, they show an increase in their fasting EGP, which may be due to glucagon-induced glycogenolysis or gluconeogenesis. This finding is consistent with the data presented by Gormsen et al [6].

The metformin-associated increase in EGP demonstrates that the mechanism of action of metformin is not entirely explained by a reduction in hepatic gluconeogenesis. However, these metformin response pathways are not necessarily mutually exclusive. In the context of marked hyperglycaemia, such as in individuals with poorly controlled diabetes, the metformin-associated increase in glucose clearance may not be sufficient to induce hypoglycaemia or stimulate counter-regulatory mechanisms, such as glucagon. In this setting, when baseline hepatic glucose output is high, the hepatic effects of metformin to reduce gluconeogenesis may dominate. A previous clamp study on the effect of metformin effect on glucose metabolism recruited individuals with poorly controlled diabetes (baseline HbA_1c_ 84 mmol/mol [9.8%]) [3]. These study participants were hyperglycaemic and had elevated EGP. It is interesting that metformin augmented non-insulin mediated glucose uptake in both our study and that by Gormsen et al, but this would have a small impact and, in the context of hyperglycaemia, would be unlikely to trigger a counter-regulatory response. However, in the healthy individual with normoglycaemia, increased glucose clearance may put the individual at risk hypoglycaemia, thereby stimulating glucagon secretion. The inhibitory effect of metformin on gluconeogenesis would be outweighed by the stimulatory effects of glucagon on glycogenolysis and gluconeogenesis.

In summary, the effect of metformin on glucose metabolism appears to be dependent on the glycaemic state of the individual. Using an alternative study design to that used by Gormsen et al, our data lends support to the suggestion that metformin increases glucose clearance, which results in an increase in EGP in the healthy individual, via counter-regulatory hormones, which is in contrast to the reduction in EGP seen in the individual with poorly controlled diabetes.

## Data Availability

The datasets generated during and / or analysed during the current study are available from the corresponding author on reasonable request.

## Abbreviations

AMPK: 5′ AMP-activated protein kinase
EGP: Endogenous glucose production
MMT: Mixed-meal test
